# Dyspnoea with progressive “idiopathic” pulmonary fibrosis

**DOI:** 10.1002/rcr2.800

**Published:** 2021-06-06

**Authors:** Chahat Puri, Vignesh Harish, Kyunghoon Rhee, Donald F. Slack

**Affiliations:** ^1^ Department of Internal Medicine Greater Baltimore Medical Center Towson MD USA; ^2^ Department of Pulmonary Medicine and Critical care Greater Baltimore Medical Center Towson MD USA

**Keywords:** Familial pulmonary fibrosis, interstitial lung disease, short telomeres

## Abstract

Telomeres are repetitive nucleotide sequences that prevent chromosomal shortening in cell replication. Short telomeres have been implicated in the pathogenesis of interstitial lung disease. Patients with short telomere related pulmonary fibrosis can have computed tomography (CT) findings inconsistent with pro‐typical usual interstitial pneumonia/idiopathic pulmonary fibrosis (UIP/IPF) pattern. They can have rapid progression and overall worse prognosis. Antifibrotic drugs, like pirfenidone, can be used to slow the progression of disease, but there is conflicting data in patients with Telomerase reverse transcriptase/Telomerase RNA component (TERT/TERC) mutations, hence genetic testing plays an important role in determining the therapeutic options. These patients should be referred for lung transplantation early. We present a case of rapidly progressive pulmonary fibrosis associated with short telomere.

## Graphical abstract

Short telomeres have been associated with familial pulmonary fibrosis. Genetic testing is essential to determine therapeutic options and perform appropriate screening

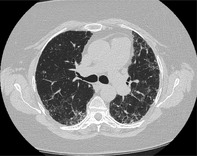

## Introduction

Idiopathic pulmonary fibrosis (IPF) is a chronic progressive idiopathic interstitial pneumonia that can sometimes occur in families. Cases of familial pulmonary fibrosis (FPF) have been linked to TERC/TERT mutations. Here, we present a case of FPF with short telomeres that had a rapid progression and a fatal outcome.

## Case Report

**Figure 1:**
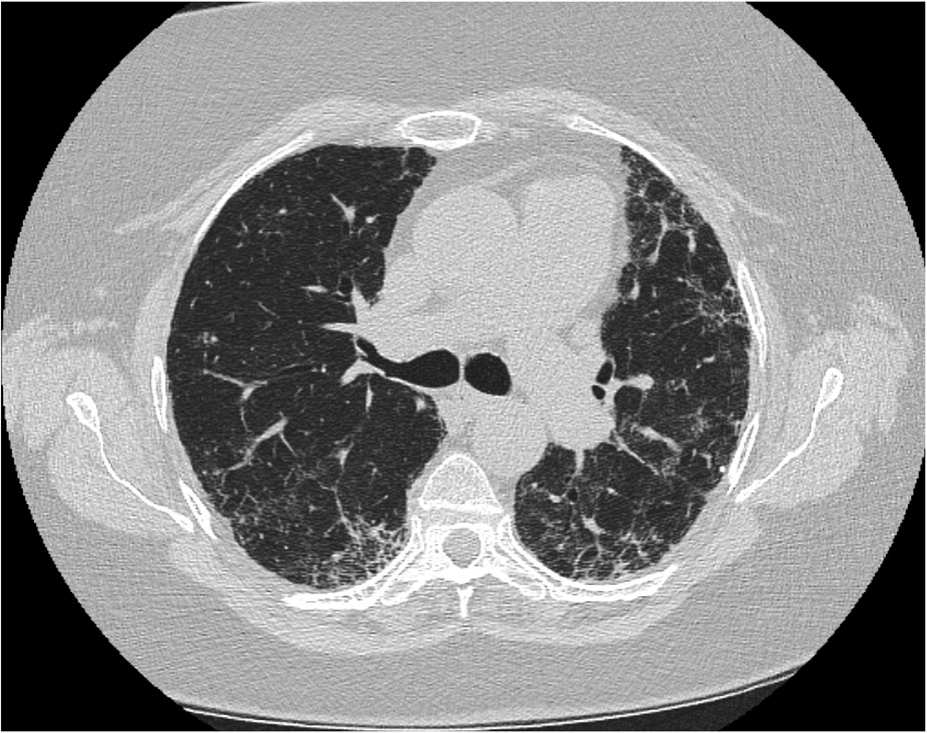
Computed tomography (CT) scan (axial section) showing fibrotic changes in the posterior and basilar part without any honeycombing.

**Figure 2:**
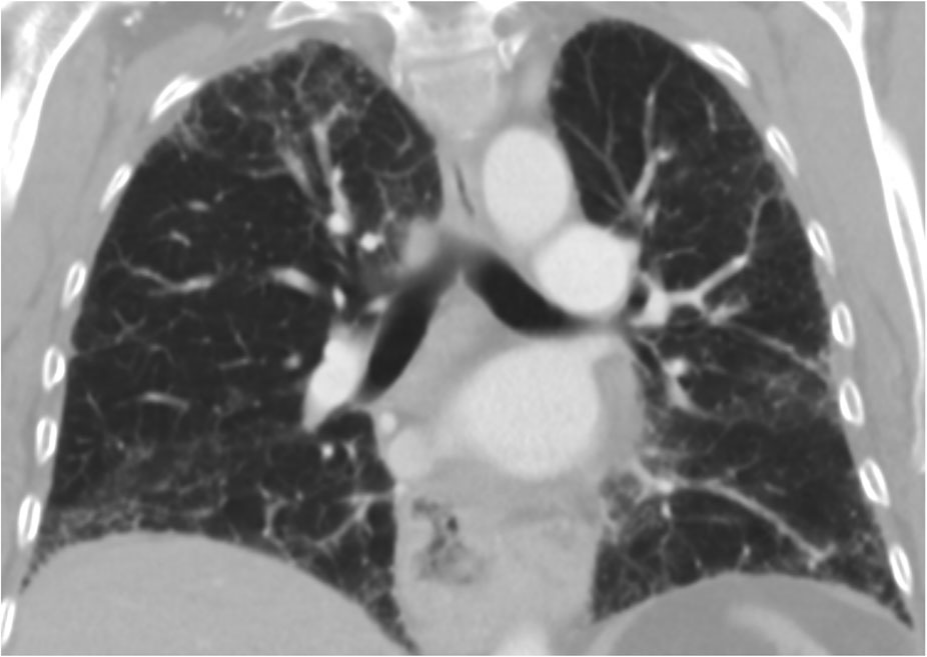
Computed tomography (CT) scan (coronal section) showing fibrotic changes in the posterior and basilar part without any honeycombing.

**Figure 3:**
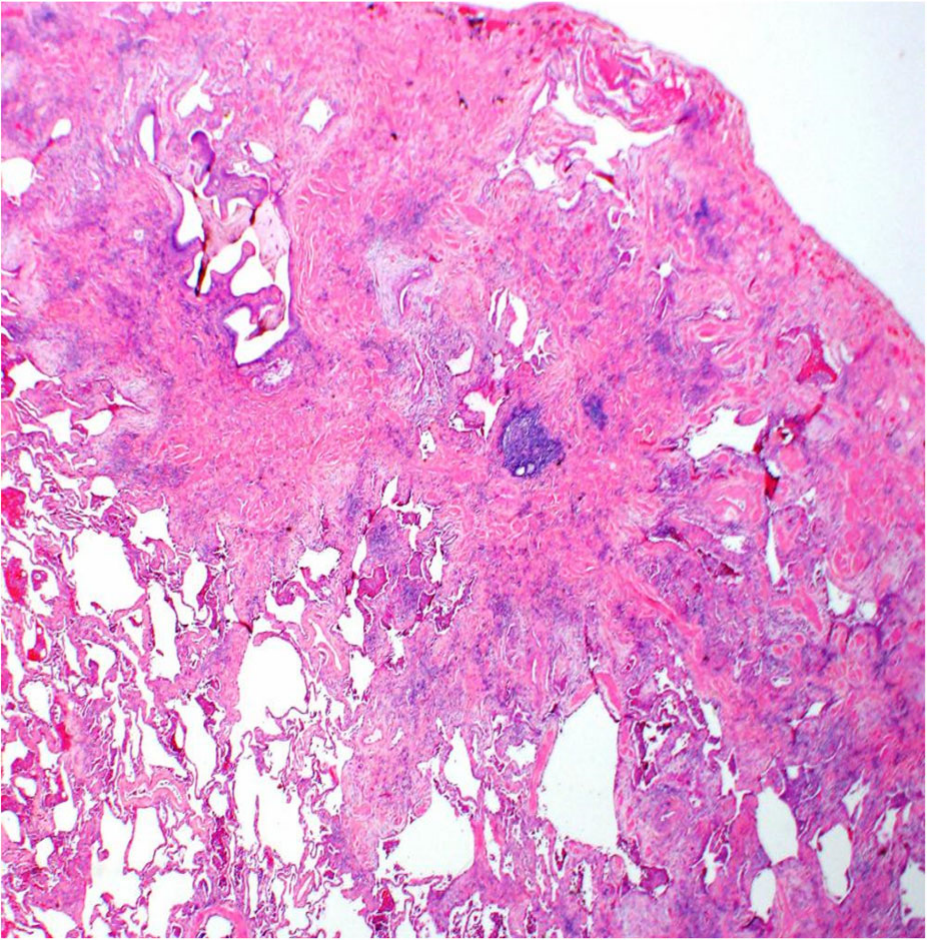
Biopsy showed patchy interstitial fibrosis with areas of normal lung architecture.

**Figure 4:**
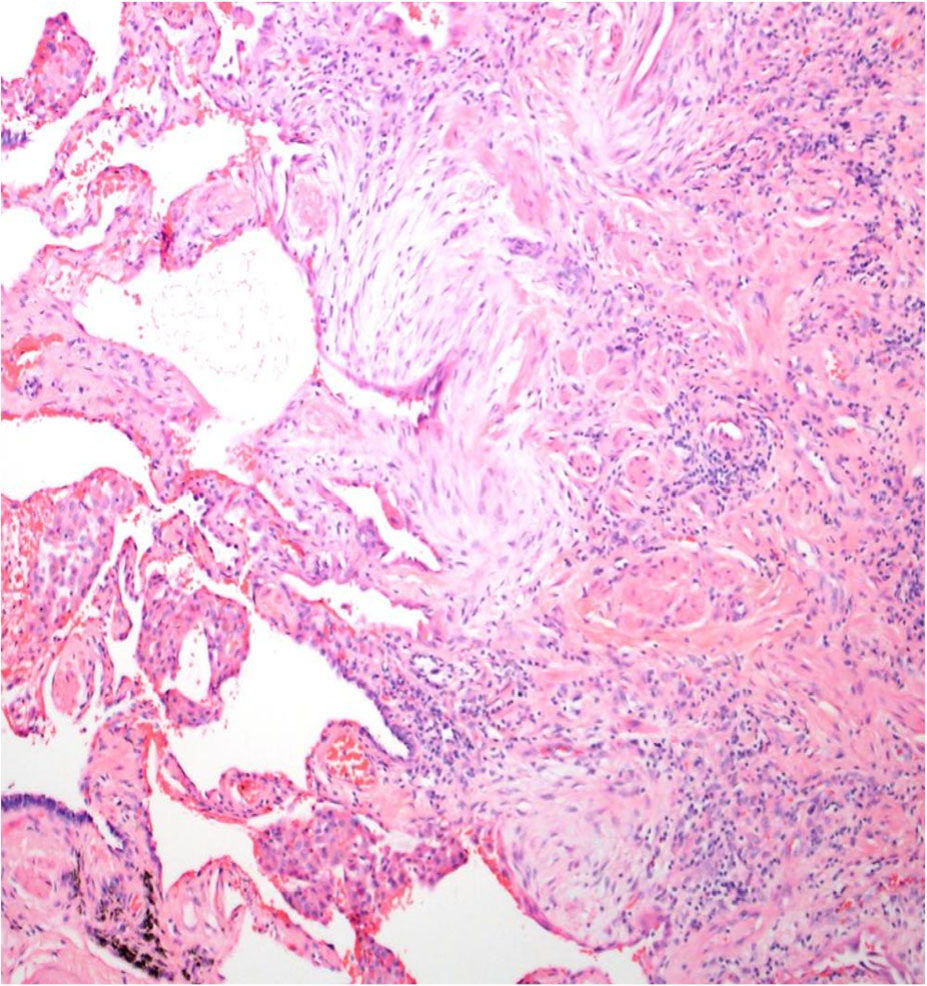
Fibroblastic foci with dense collagen fibrosis.

A 63‐year‐old female with a past medical history of hypertension, hypothyroidism, and mild intermittent asthma presented to her primary care physician with dyspnoea and a progressive dry cough. It started two weeks prior to presentation, without associated fevers, chills, chest pain, or weight changes. She had a 10‐pack year smoking history but no other exposure to any workplace or environmental toxins or chronic medications known to have pulmonary toxicity. Her family history was significant for an identical twin who was diagnosed with hypersensitivity pneumonitis (HP) and her mother was diagnosed with IPF. There was no family history of cirrhosis or premature greying of hair. She was started on inhalers and prednisone for presumed bronchitis but returned two weeks later with worsening shortness of breath, extreme fatigue, and progressive exercise intolerance. Her vitals were normal, and the physical examination showed normal S1 S2, with no murmurs. Lung examination revealed fine bibasilar inspiratory crackles, with no wheezing. She did not have any palpable lymphadenopathy, rashes, joint tenderness, or small joint deformities. Chest X‐ray was performed that showed bilateral interstitial fibrosis. Complete blood count and liver function tests were normal. C‐reactive protein (CRP) was elevated at 6.13 mg/dL and erythrocyte sedimentation rate (ESR) 92 mm/h. Immunological work‐up was negative, except anti‐nuclear antibody (ANA) 1:160 (Table 1). Chest computed tomography (CT) (Figs. 1, 2) demonstrated fibrotic changes of bilateral lungs with posterior and basilar predominance without honeycombing. Pulmonary function tests (PFTs) showed forced expiratory volume in the first second (FEV_1_) of 1.42(75% predicted), forced vital capacity (FVC) 1.72 (66% predicted), total lung capacity (TLC) 2.23 (53% predicted), and diffusion lung capacity for carbon monoxide (DLCO) of 5.3 (25% predicted) consistent with severe restriction. Repeat PFTs one month later showed worsening FEV_1_ of 0.93 (43% predicted) and TLC of 1.75 (40% predicted) consistent with restrictive physiology. Given the rapid progression, she had a lung biopsy consistent with usual interstitial pneumonia (UIP) (Figs. 3, 4) and subsequent telomere tests done demonstrated lymphocyte telomere length less than the 10th percentile for age confirming the diagnosis of short telomere related pulmonary fibrosis. She was being evaluated for a lung transplant, but unfortunately she passed away within three months of her diagnosis.

**Table 1:** Summary of immunological work‐up for ILD.

Test	Value	Reference range
ANA	1:160	<1:40
Ro52	<2.3	<20 units
Ro60	<4.9	<20 units
ds‐DNA	Negative	Negative
ANCA	Negative	Negative
PR3	<2.3	<20 units
CCP	8.9	<20 units
RNP/Smith	<3.3	<20 units
Ig panel	Negative	Negative
PL‐12 AB	<11	<11 units
PL‐7 AB	<11	<11 units
EJ AB	<11	<11 units
Anti‐Jo/Jo‐1 AB	<11	<11 units
MI‐2 BETA AB	<11	<11 units
MI‐2 ALPHA AB	<11	<11 units
SRP AB	<11	<11 units
NXP‐2 AB	<11	<11 units
TIF‐1y AB	<11	<11 units
MDA‐5 AB	<11	<11 units
Centromere AB	Negative	Negative
C3/C4	Normal	

ILD, Interstitial lung disease.

## Discussion

Telomeres are repetitive nucleotide sequences that prevent chromosomal shortening during cell replication. Mutations in the telomerase enzyme activity, telomerase biogenesis, maturation, and trafficking can result in the shortened telomere.

Short telomeres can result in severe multisystem disorders, known as short telomere syndrome like dyskeratosis congenita. Patients with this disorder usually present with mucocutaneous abnormalities such as mucosal leukoplakia, nail dystrophy, premature greying of hair, bone marrow failure, increased risk of skin cancer, IPF, and rarely cirrhosis [1].

Short telomeres have been implicated in the pathogenesis of interstitial lung disease. The common patterns seen are fibrosis, nonspecific interstitial pneumonia (NSIP), organizing pneumonia, HP, acute interstitial pneumonia, and combined fibrosis and emphysema. TERT and TERC gene mutations have been associated with FPF [2]. Studies have shown that about 30% of patients with FPF have telomere‐related mutations [3].

Not all patients with ILD secondary to short telomere develop FPF. Our patient had a CT scan inconsistent with the prototypical UIP/IPF pattern but histological evidence of UIP. In studies involving FPF with telomere mutations, more than half of the patients with CT findings inconsistent with IPF had biopsy‐proven UIP [2]. This was the case in our patient as well. Her twin sister's images were most consistent with chronic HP.

Patients with short telomere related ILD progress rapidly and have a worse prognosis. Identified patients should be referred for lung transplantation immediately. Antifibrotic agents, nintedanib and pirfenidone, limit disease progression; however, there is conflicting data to suggest that patients with TERT/TERC mutations are less likely to respond to pirfenidone [4]. More prospective trials are needed to establish the efficacy of these medications. There is inconclusive evidence for the use of danazol in patients with IPF. It has been shown to cause telomere elongation and slow the decline in FVC and DLCO; however, there are case reports of worsening fibrosis after the initiation of danazol [5]. Given the inconclusive evidence, the use of danazol in short telomere related ILD is currently restricted to patients with aplastic anemia. There is also ongoing research for gene therapy in the management of patients with familial ILD. Telomere mutations must be identified in patients with IPF undergoing transplant as their immunosuppressant therapy will be tailored accordingly due to increased toxicity. Genetic counselling should be done for immediate family members and telomere testing should be done even if specific IPF pattern is not seen on CT scans.

### Disclosure Statement

Appropriate written informed consent was obtained for publication of this case report and accompanying images.

### Author Contribution Statement

All authors contributed to the care of this patient and were involved in writing the manuscript. Chahat Puri wrote the main manuscript. Kyunghoon Rhee and Vignesh Harish helped in writing the abstract and case presentation. Donald F. Slack made all the edits and helped with the discussion and obtained the clinical images.
